# “Putting yourself in the shoes of others” – Relatability as a novel measure to explain the difference in stigma toward depression and schizophrenia

**DOI:** 10.1007/s00127-024-02807-x

**Published:** 2024-12-23

**Authors:** Georg Schomerus, Johanna Kummetat, M. C. Angermeyer, Bruce G. Link

**Affiliations:** 1 Department of Psychiatry and Psychotherapy, University of Leipzig Medical Center, Semmelweisstr. 10, 04103 Leipzig, Germany; 2 Center for Public Mental Health, Gösing am Wagram, Austria; 3 Department of Sociology, University of California Riverside, Riverside, USA

**Keywords:** Stigma, Empathy, Continuum beliefs, Schizophrenia, Depression, Psychopathology

## Abstract

**Purpose:**

Attitudes toward schizophrenia and depression have evolved differently over the last decades, exposing people with schizophrenia to growing stigma. Classic descriptions of schizophrenia symptoms as being particularly unrelatable might offer an explanation for this gap in attitudes that has not yet been tested. We examine to what extent relatability explains the difference in social distance toward people with depression or schizophrenia.

**Methods:**

We developed the 8-item “Relatability Scale”, measuring to what extent people can relate to someone described as having either depression or schizophrenia, and used it in an online quota sample of 550 respondents in Germany. Beyond, we elicited the desire for social distance, continuum beliefs, emotional reactions, perceived dangerousness, general empathy, and previous contact.

**Results:**

The Relatability Scale showed good psychometric properties and construct validity. Differences in relatability alone explained 63.6% of this difference in social distance between depression and schizophrenia. Adding continuum beliefs increased this amount to 83.0%. All other variables combined explained 53.2% of the difference in social distance between disorders.

**Conclusion:**

Differences in both relatability and continuum beliefs seem key to understanding different reactions to someone with depression or schizophrenia. Anti-stigma interventions could be optimized in order to increase relatability and continuum beliefs particularly regarding people with severe, psychotic mental disorders.

**Supplementary Information:**

The online version contains supplementary material available at 10.1007/s00127-024-02807-x.

## Introduction

Attitudes toward people with mental illnesses have changed over the last decades. Trend studies among the general population that use vignettes, i.e. descriptions of people with a mental illness as a stimulus, consistently show a widening gap between attitudes towards someone with schizophrenia and someone with depression [1–4]. While attitudes towards someone with depression appear to have improved, even seeming to align with attitudes towards a clinically healthy “troubled person” in the U.S [4]., attitudes towards schizophrenia are deteriorating: The desire for social distance toward people with schizophrenia is growing [2–4]. Moreover, people with schizophrenia provoke less sympathy, a reduced desire to provide help [3], their symptoms are less likely to be seen on a continuum with normal experiences [5], they are more frequently regarded as threatening and violent, and involuntary hospitalization is more readily endorsed [6] – all differences that, although present since they have first been examined, have increased in magnitude over the last decades. This widening gap between schizophrenia and depression in the eyes of the public has been found in the two longest-running trend studies, in Germany and in the United States [3, 4, 7], both being conducted in large population samples, and using identical sampling strategies, survey methodology, item wording and wording of the case vignettes over the entire study period. Since changes in social distance differ between disorders, they are unlikely to represent broad changes in general attitudes towards mental illness, but seem to be illness specific. To prevent this gap from widening further, and to address the stigma of severe, psychotic mental illness in particular, it is urgent to understand what makes perceptions of depression and schizophrenia so different, and whether this difference is potentially responsive to targeted interventions.

A fundamental difference, a kind of watershed notion that differentiates among psychiatric disorders was postulated in 1946 by German psychiatrist Karl Jaspers, who emphasized the strange, unfamiliar nature of some mental disorders in order to define and distinguish broad categories of mental illness. Specifically, he used the terms “Gemütskrankheiten” versus “Geisteskrankheiten” (disorders of the mind versus insanity) to separate psychotic disorders like schizophrenia from of more relatable mental disorders like depression: “The most profound difference in mental life seems to be between states that we can empathize with, that are understandable, and schizophrenic mental life that is in its own way incomprehensible and, in the true sense, crazy.” [8]. In fact, Jaspers is often cited to describe the “exceptionally unusual nature of the experiences” associated with schizophrenia [9].

Interestingly, the same themes of incomprehensibility were brought forward by Shirley Star [10] in the first nationwide study of public attitudes toward mental illnesses in the United States (see [11] for context). Seeking to understand how members of the public decided whether a pattern of behavior was a mental illness, Star emphasized that people came to the judgement when they could not explain the behavior any other way, when it lacked reason, was out of control and was incomprehensible to them. When such a judgement was made, people, according to Star, were propelled to want to keep mental illness as “far from themselves as possible” [10].

Several decades later Rosenberg, the developer of the often-used self-esteem scale, took up the same question as Star but from the vantage point of so-called symbolic interactionism within sociology [12]. This perspective claims that smooth and effective social interaction derives from one individual’s ability to put themselves in the role of the other, anticipating the other’s reaction and thereby guiding their social behavior. From this vantage point Rosenberg derived the proposition regarding public assessments of mental illness that “the defining feature of psychosis is the observer’s inability to take the role of the actor.” As he explained it, this inability “is totally destructive of meaningful human interaction, which requires a person to anticipate the others´ response to his or her words and actions. But if we have no comprehension of what is going on in the other’s mind (…) then the human bond is snapped.”

Applied to the vignette studies on depression and schizophrenia, we assume that differences in public assessments of how *relatable*, as opposed to incomprehensible, these disorders appear, can explain the apparent difference in social distance. If the experience of having a disorder is more difficult to relate to, people might be less inclined to engage in interactions with a person having such a disorder and therefore desire greater social distance from them. We use the term “relatability” (and prefer it over “perspective taking”, for example), since it describes both the perceived difference between *mental disorders*, which are supposedly relatable to different degrees, and differences in the ability of *respondents* to relate to someone with these disorders.

Another construct that has been examined with regard to the perceived “otherness” of people with mental illness are continuity beliefs [13]. If the degree of relatability someone expresses toward a person with a mental disorder can be viewed as *bridging a gap* between “us” und “them”, continuum beliefs describe the *perception of this gap*: Is there a fundamental difference between mental health and mental illness, between “us” and “them”, people with and without mental disorders? Or is there a gradient that puts everybody on the same continuum of symptoms experiences, where it is only a matter of severity, frequency and duration whether we cross the threshold for a formal diagnosis? Numerous correlational studies have shown that belief in a mental health-mental illness continuum is associated with less social distance for a wide range of mental disorders like depression, schizophrenia, alcohol use disorder and obsessive compulsive disorder [14], and some experimental studies even suggest a causal relationship [15, 16]. Hence, relatability and continuum beliefs are allied concepts, addressing the “differentness” of people with and without mental disorders from two distinct angles. Since continuum beliefs have been shown to evolve differently over the last decade, increasing for depression and declining for schizophrenia [5], we include them into our study as another variable that might explain the growing difference in reactions towards depression and schizophrenia.

In sum, diverging trends in attitudes toward depression and schizophrenia posed a critically important question that pushed us to return to several classic statements within psychiatry that might help answer such a question. Using an online survey among people living in Germany, we want to find out whether, and to what extent, both relatability and continuum beliefs explain the difference in reactions to a vignette depicting either a person with depression or with schizophrenia. Furthermore, we want to examine whether relatability explains the difference in social distance towards people with depression and schizophrenia beyond other constructs, like emotional reactions and perceptions of dangerousness, that have been shown to be related to social distance and to differ between the two disorders [17].

## Methods

### Sample

We conducted an online survey among people living in Germany (18 years and older, *n* = 550), using an established online access panel with quota sampling, with quotas for age, gender, and state proportional to the general population in Germany. Details of the quota sampling procedure are given in the online supplement.

### Vignettes

At the beginning of the survey, we presented respondents with a case vignette depicting either a person with schizophrenia or major depression. The gender of the person (Lisa, Chris) varied at random. All vignettes started with “Imagine that you learn the following about [Lisa] who lives near you”, followed by a description of [Lisa] suffering from loss of energy, loss of interest, anhedonia, low mood, hopelessness, sleeping problems, social withdrawal and difficulties in daily activities (depression, see appendix for the wording of the vignettes), or from paranoid delusions, auditory hallucinations, thought control and thought broadcasting, social withdrawal and strong anxiety (schizophrenia, see appendix). The vignettes were constructed to be almost identical to the vignettes used in the German stigma study 1990–2020 [3], the main difference being that the described person was given a name to enhance potential relatability with the character.

### Development of the relatability scale

Since there is no scale assessing relatability with regard to a person with mental illness, we constructed a relatability scale for this purpose. We formulated an initial set of 18 different items, which we discussed and reduced to nine by removing redundant items. Among these nine items, four affirmed relatability (“It is easy for me to put myself in the situation [Lisa] is experiencing”), and five were phrased reversely (“Her behavior appears to me completely strange and incomprehensible”). Answers were given on a five-point Likert scale with anchors 1 (“don’t agree at all”) to 5 (“agree completely”). Pretesting these items in 30 respondents prior to the online study confirmed that items were understandable and easy to answer.

Examining internal consistency of the scale in the full sample in the online study, one item showed a low item-test correlation (0.52) compared to the other items (0.72–0.80). Excluding this item resulted in a scale with four positive and four negatively framed items and an overall alpha of 0.90 (Table 1). Our representation of the items in a single scale was supported by an exploratory factor analysis that produced an eigenvalue of 4.22 for a first factor followed by one of 0.74. Given the sharp drop off of the eigenvalues after the first factor and a second factor with an eigenvalue less than one led us adopt a single factor solution. We use the mean value of the scale (1–5) with higher values indicating greater relatability.

**Table 1:** Relatability Scale Item Characteristics

	“Please carefully read the following statements and mark your response. Please indicate your personal opinion. There is no right or wrong answer.”	Total *n* = 550 M (SD)	Schizophrenia *n* = 275 M (SD)	Depression *n* = 275 M (SD)	Item-test corr.
1	It is easy for me to put myself in the situation that [Lisa] is experiencing.	3.32 (1.16)	2.96 (1.06)	3.68 (1.04)	0.74
2	Her behavior appears to me completely strange and incomprehensible. (R)	2.63 (1.22)	3.04 (1.16)	2.23 (1.15)	0.78
3	From my perspective her behavior doesn’t make any sense. (R)	2.50 (1.18)	2.85 (1.17)	2.15 (1.07)	0.78
4	I can understand why [Lisa] is the way she is.	3.30 (1.08)	2.96 (1.09)	3.64 (0.96)	0.76
5	It is easy for me to appreciate what it would be like to be in [Lisa´s] situation.	3.24 (1.21)	2.87 (1.23)	3.60 (1.08)	0.76
6	There is no reason to behave like she does. (R)	2.46 (1.19)	2.77 (1.23)	2.15 (1.07)	0.77
7	I can empathize with how [Lisa] feels in this situation.	3.50 (1.11)	3.17 (1.13)	3.84 (0.98)	0.76
8	There is no way I can understand why [Lisa] behaves and feels the way she does (R)	2.36 (1.14)	2.66 (1.16)	2.05 (1.04)	0.76

Note. ***M***: mean, ***SD***: standard deviation

### Continuum beliefs

We assessed continuum beliefs addressing two different aspects of a continuity between mental health and mental illness. The first included two items addressing a continuity between a respondent themselves and the person described in the vignette (*Us versus them continuum beliefs*: “Sometimes, we are all like Lisa, it’s just a question of how pronounced this state is”; “In extreme circumstances, many of us could show signs of [Lisa’s] problems.”, alpha 0.75). The second included two items addressing a conceptual continuity between mental health and mental illness (*General continuum beliefs*: “There is a clear boundary between mental illness and mental health” (R); “Mental health and mental illness are separate and completely different conditions” (R), alpha 0.76). These items were all answered on five point Likert scales from 1 (“don’t agree at all”) to 5 (“agree completely”), resulting in mean scores from 1 to 5 [14]., with higher scores indicating stronger continuum beliefs.

### Social distance

We used the Social Distance Scale by Link and co-workers [18]. Respondents answered eight items like: “Would you accept someone like [Lisa] as a co-worker?” on a five point Likert-scale with anchors 1-“definitely” to 5 “definitely not”. Internal consistency of the scale is high (alpha 0.88 in our sample). For our analysis, we divided the sum-score of the scale by the number of items, resulting in a mean score from 1 to 5, higher scores indicating a greater desire for social distance.

*Other measures* include the Emotional Reactions towards Mental Illness (ERMIS) scale [19], measuring anger, fear and prosocial reactions toward the vignette character. Again we used the mean score for each subscale, ranging from 1 to 5, with higher values indicating stronger emotional reactions. We used an item from the General Social Survey on perceived dangerousness [6], yielding scores from 1 (low perceived dangerousness) to 4 (high perceived dangerousness). Previous contact was assessed as 0 = no contact, 1 = knows someone; works together or works with, 2 = has a friend/family member with mental illness, 3 = has experienced mental illness themselves. We further used the German version of the Toronto Empathy Questionnaire (TEQ-D) [20], yielding mean scores from 0 to 4, higher scores indicating greater empathy. Details on these measures and scoring can be found in the online supplement.

### Analysis

We conceptualized relatability, continuum beliefs and social distance as continuous variables. We used pairwise correlation analysis to test the construct validity of the newly generated relatability scale. Since correlation analysis showed that both relatability and social distance were much more closely associated with us/them continuum beliefs than with general continuum beliefs (*r* =.55 vs. *r* =.14 and *r* =.48 vs. *r* =.17, respectively, see Table 2 and below), we focussed our analyses on us/them continuum beliefs, using general continuum belies as an additional potential confounder in our multivariate models.

In order to assess whether relatability, and us/them-continuum beliefs, might explain why people want more social distance from a person with schizophrenia than from a person with depression, we implement four sequentially ordered analyses. To begin, we examine whether there is a substantial and significant difference in expressed social distance toward a person with schizophrenia compared to a person with depression. Second, we test whether responses to our newly created measure of relatability and us/them continuum beliefs are lower for people assigned the schizophrenia vignette as compared to the depression vignette. Third, we test whether relatability and us/them continuum beliefs are significantly independently associated with social distance. Fourth, we examine whether relatability and us/them continuum beliefs account for any of the association between vignette type and social distance and if so how much. Here we gauge the importance of both constructs by quantifying how much of the social distance gap between schizophrenia and depression relatability and continuum explain, both independently and combined, also adding the other potentially confounding variables to the model. For multivariate analyses, we used ordinary least squares regression. We used the Stata supported program of Kohler and Karlson [21] to quantify the proportion of the vignette (schizophrenia versus depression) to social distance association that was explained by relatability and continuum beliefs, and to test the significance of the proportion explained (the indirect effect).

## Results

### Construct validity of the relatability scale

Table 2 shows the correlation of the Relatability Scale with measures related to the vignette character (upper half) and with general attitudes unrelated to the vignette (lower half). Relatability correlates as expected with every measure we tested it against. The strongest correlation involves us/them continuum beliefs about the vignette character (0.55) suggesting that the higher the relatability score, the more respondents also believe that all of us could sometimes be like [Lisa]. The lowest correlation, but still significant in the expected direction, is between relatability and general continuum beliefs, not about the vignette but mental illness in general (0.14). We conclude that there is some evidence for construct validity. At the same time we also note that some of the associations are sizeable, a fact that leads us control these associated variables in our main hypothesis tests.

**Table 2:** Correlations of Relatability with other Constructs

	Correlation
**Measures related to vignette character**	
Emotional Reactions Anger Fear Prosocial	− 0.50 *** − 0.27 *** 0.46 ***
Continuum Beliefs – “Us versus them”	0.55 ***
Dangerousness	− 0.27 ***
**Measures unrelated to vignette character**	
Contact	0.28 ***
Empathy	0.43 ***
Continuum beliefs - general	0.14 ***

Note. ***p <.001; Emotional Reactions: Anger (feel angry, annoyed, incomprehension); Fear (they scare me, make me feel insecure, uncomfortable) and Prosocial (I feel pity, sympathy, want to help them). Empathy: TEQ-D, 16-item scale; Contact: 0 = no contact, 1 = know someone, work together, 2 = friend, family, 3 = self

### Difference in social distance toward someone with schizophrenia or depression

As expected, a vignette person with schizophrenia elicited more social distance than a vignette person with depression (Mean, 3.31 vs. 2.82, Effect Size (ES) 0.58, *p* <.001). This gap was similar for the female vignettes (ES 0.61) and male vignettes (ES 0.54, both *p* <.001).

### Differences in relatability and continuum beliefs regarding someone with schizophrenia or depression

Mean relatability toward a vignette person with depression was 3.77, toward a person with schizophrenia 3.08, (ES 0.76, *p* <.001). Again, this difference was significant both for the male character (ES 0.63, *p* <.001) and the female character (ES 0.94, *p* <.001). Mean us/them continuity beliefs were 3.71 for depression, and 2.96 for schizophrenia (ES 0.79, *p* <.001).

### Relatability, continuum beliefs, and desire for social distance

**Fig. 1:**
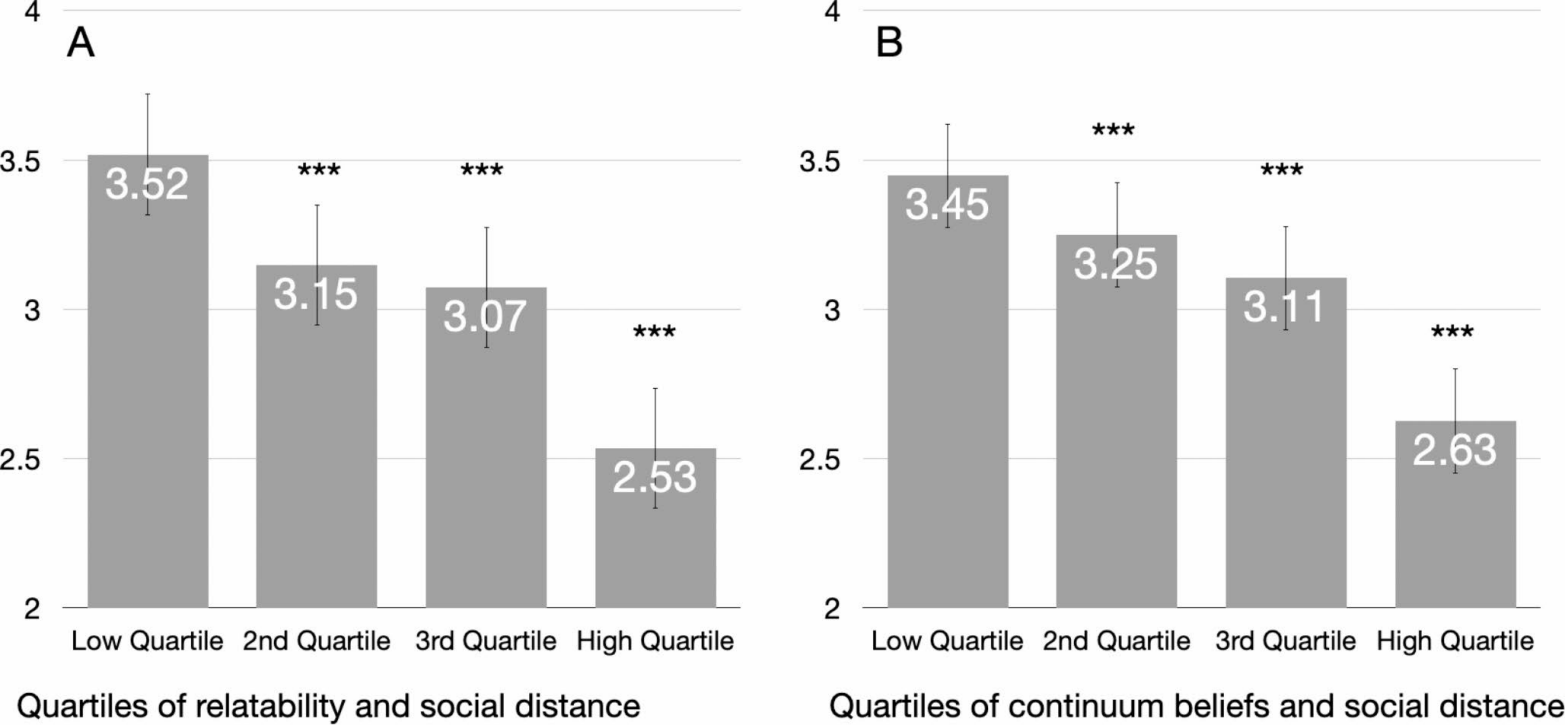
Desire for Social Distance and Quartiles of Relatability (**A**) and Us/Them-Continuum Beliefs (**B**) Means of social distance, adjusted for vignette characteristics (disorder type and gender). *** *p* <.001 compared to lowest quartile. Error bars represent one standard error

Social distance was correlated − 0.51 (*p* <.001) with relatability. Regarding continuum beliefs, us/them continuum beliefs showed a much stronger correlation with social distance than general continuum beliefs (-0.48 versus − 0.17). We thus focussed our analyses on relatability and us/them continuum beliefs. To visualize the associations, Fig. 1 shows means of social distance by quartiles of relatability (panel A) and us/them continuum beliefs (panel B), adjusted for disorder and gender of the vignette. As the figure shows, effect sizes comparing social distance in the lowest quartile to the highest quartile of relatability (Panel A) and us/them continuum beliefs (Panel B) were 1.13 (*p* <.001) and 0.85 (*p* <.001) respectively. Respondents most able to relate to the person described and those most likely to view the described person on a continuum with themselves were substantially less likely to desire social distance.

### Can variables other than relatability and us/them continuum beliefs explain the association between vignette disorder and social distance?

Table 3, Eq. 1 shows associations between social distance and vignette disorder type (schizophrenia versus depression) and vignette gender. Random assignment to the schizophrenia vignette is associated with a stronger desire for social distance (b = 0.489, *p* <.001), random assignment to a female vignette associated with a slightly lower desire for social distance (b = − 0.166, *p* <.05). Equation 2 adds respondent sociodemographic characteristics and shows that only age is significantly associated with social distance, with older people indicating a greater desire for distance (b = 0.005, *p* <.01). As respondent characteristics are largely balanced by randomization, adding these demographic variables did not, as expected, account for a significant proportion (4.1%, n.s.) of the vignette disorder to social distance association. Equation 3 adds two additional respondent characteristics, prior contact with people with mental illnesses and a personal orientation to empathy, each of which is strongly associated with lower social distance (b=-0.154, *p* <.001; and b=-0.442, *p* <.001, respectively). This time, the proportion of the vignette disorder to social distance association accounted was relatively small (12.4%) but nevertheless statistically significant (*p* <.05). Equation 4 adds variables assessing respondents’ perceptions about the vignette character, many of which have been used in prior research to understand why people might desire social distance from the vignette characters. As Eq. 4 shows, emotional reactions of fear (b = 0.274, *p* <.001), anger (b = 0.111, *p* <.05), and prosocial (b=-0.348, *p* <.001), as well as general continuum beliefs (b=-0.066, *p* <.05) were all independently related to social distance. Perceptions of the dangerousness of the vignette character was the only added variable not significantly related to social distance, likely because emotional reactions were controlled (entered by itself to variables in Eq. 2, dangerousness is significantly associated with social distance in the expected direction). However, the most important findings from Eq. 4 were (1) that even with all of these variables entered, only slightly more than half (52.4%) of vignette disorder to social distance association was accounted for and (2) the vignette disorder variable remained robustly significant (b = 0.234, *p* <.001). Hence, these variables do not fully explain why people react differently to vignettes depicting depression or schizophrenia.

**Table 3:** Ordinary Least Squares Regression Assessing whether Multiple Strong Predictors of Social Distance from Prior Research Can Account for the the Association between Vignette Disorder Type and Social Distance (*N* = 550)

	Equation 1.	Equation 2	Equation 3	Equation 4
Vignette Disorder (Schizophrenia = 1; Depression = 0)	0.489*** (0.070)	0.469*** (0.069)	0.429*** (0.065)	0.234*** (0.061)
Vignette **Gender** (Female = 1; Male = 0)	− 0.166* (0.070)	− 0.172* (0.069)	− 0.188** (0.065)	− 0.067 (0.058)
Respondent **Gender** (Female = 1; Male = 0)		− 0.138 (0.071)	0.034 (0.069)	− 0.027 (0.061)
Respondent Age (Years)		0.005** (0.002)	0.005* (0.002)	0.005** (0.002)
Education High (qualifications 12/13 years = 1; basic 9 years = 0) Medium (10 years = 1; Basic 9 years = 0)		0.101 (0.099) 0.059 (0.099)	0.161 (0.093) 0.095 (0.093)	0.204* (0.085) 0.110 (0.083)
Contact with People with Mental Illnesses			− 0.154*** (0.031)	− 0.116*** (0.028)
Toronto Empathy Scale			− 0.442*** (0.068)	0.055 (0.082)
Emotional Reactions Fear				0.274*** (0.038)
Anger				0.111* (0.052)
Prosocial				− 0.348*** (0.046)
General Continuum Beliefs				− 0.066* (0.029)
Perceptions of Dangerousness				− 0.021 (0.045)
Significance of indirect effect from Vignette Disorder to all variables added in each equation to Social Distance (KHB)		n.s.	*	***
Percent of Vignette Disorder to Social Distance association accounted for by all added variables (KHB)		4.1%	12.4%	52.4%
R-square	9.1%	11.4%	22.8%	40.5%

Note. Regression coefficients (standard errors). ***p <.001, **p <.005, *p <.05

### Relatability and us/them continuum beliefs as potential mediators of the association between vignette disorder (schizophrenia or depression) and desire for social distance

Table 4 shows that adding relatability to a regression analysis predicting the desire for social distance (with vignette disorder and vignette gender as independent variables, Eq. 2) increases the amount of variance explained from 9.1 to 27.3%. There is a highly significant indirect effect between vignette disorder and social distance, which accounted for 63.6% of the association between vignette disorder and social distance. Equation 3 tested whether us/them continuum beliefs might also account for the relationship between vignette disorder and social distance. In fact, there is also a highly significant indirect effect, accounting for 58.3% of the vignette disorder to social distance association. The explained variance of this model is 24.6%, thus only slightly less than the model (Eq. 2) containing relatability. When adding both us/them continuum beliefs and relatability (Eq. 4), the model explains 32.1% of the variance of social distance, and the direct effect of vignette disorder on social distance is no longer significant. The indirect effects of relatability and continuum beliefs accounted for 83.0% of the association between vignette disorder and social distance. Equation 5 adds all of the variables assessed in Table 4, including prior contact, the Toronto empathy scale, general continuum beliefs, perceptions of the dangerousness and emotional reactions of fear, anger, and prosocial emotions. As Eq. 5 shows, adding all of these variables does little to further explain the vignette to disorder association – it climbs only 4.2% from 83.0 to 87.2% and both us/them continuum beliefs and relatability remain significant independent predictors of social distance. Both relatability and us/them continuum beliefs are thus sufficient to explain most of the difference in reactions to depression or schizophrenia.

**Table 4:** Ordinary Least Squares Regression Assessing the Role of Relatability and Us/Them Continuum Beliefs in Explaining the Association between Vignette Disorder Type and Social Distance (*N* = 550)

	Equation 1.	Equation 2	Equation 3	Equation 4	Equation 5
Vignette Disorder (Schizophrenia = 1; Depression = 0)	0.489*** (0.070)	0.178** (0.068)	0.204** (0.069)	0.083 (0.067)	0.066 (0.063)
Vignette **Gender** (Female = 1; Male = 0)	− 0.166* (0.070)	− 0.130* (0.062)	− 0.142* (0.063)	− 0.125* (0.060)	− 0.065 (0.056)
Relatability		− 0.448 *** (0.038)		− 0.325*** (0.042)	− 0.139** (0.045)
“Us – Them” Continuum Beliefs referring to vignette Character			− 0.378*** (0.036)	− 0.238*** (0.038)	− 0.174*** (0.035)
Significance of indirect effect Vignette Disorder to Relatability to Social Distance (KHB method)		***	***	***	***
Percent of Vignette Disorder to Social Distance association accounted for (KHB method)		63.6%	58.3%	83.0%	87.2%
R-square	9.1%	27.3%	24.6%	32.1%	45.4%

Note. Regression coefficients (standard errors). ***p <.001, **p <.005, *p <.05

## Discussion

We found that relatability explained a large proportion of the association between vignette type (depression versus schizophrenia) and social distance. When joined with the allied measure of “us versus them” continuum beliefs, the proportion of the association explained rose to 83%. These findings have implications for (1) how we understand the gap in social distance between depression and schizophrenia, (2) how we might decrease the gap by intervening to increase relatability and (3) how we might explain the results of trend studies showing a widening gap.

### Understanding differences in the desire for social distance between disorders

At first glance, our findings align with the proposed distinction between depression and schizophrenia, pointing toward relatability as a key difference between different mental disorders or states of mind. By positing “relatable” disorders against “unrelatable” ones, Jaspers, Star and Rosenberg constructed a polar difference in relatability between “Gemütskrankheiten” and “Geisteskrankheiten”, or between the “sane” and the “insane”, either from a general point of view based on psychopathology (Jaspers), or as individual or societal reactions to unpredictable behavior (Star and Rosenberg). In our study, however, the difference in reactions toward depression or schizophrenia is largely mediated by *dimensional* constructs. We used a continuous measure of relatability, and in fact, the person with schizophrenia was not perceived as completely unrelatable, but, on average, as less relatable than the person with depression. There was variance in relatability for both disorders, there were respondents more or less able to relate to the person described, and for both disorders, more relatability was associated with less social distance. Our results thus challenge a postulated categorical difference between relatable and unrelatable states of mind. Rather, they show that differences in relatability can be integrated within a continuous model of mental illness. To quote Rosenberg again, we did not find “the human bond [being] snapped”, but rather stretched, to a lesser or stronger degree. Dimensional constructs, in turn, open up the opportunity to think about how to improve the ability of people to relate to someone with a severe mental health problem. In terms of anti-stigma interventions, more relatability seems to be a much more achievable goal than total relatability.

With regard to continuum beliefs, our findings also broaden our understanding of their importance for the stigma of mental illness: our study suggests that here, the continuum between mental health and illness is of particular relevance if it is not solely framed as an abstract continuum between two poles (health and illness, or normality and abnormality, as measured with our items on general continuum beliefs), but also between “us” and “them” [14, 22]. From the angle of illness concepts, too, the willingness to personally relate one’s own experiences to the experiences of the described person seems particularly relevant.

### Interventions to increase relatability toward people with schizophrenia

Our findings further suggest that increasing the relatability of mental illness, particularly of schizophrenia, will reduce the desire for social distance from people with this condition. There are studies examining different strategies to directly promote the understanding of the experience of having schizophrenia, but with mixed effects. For example, simulations of either auditory or visual hallucinations showed mixed, and sometimes counter-productive effects on empathy, stereotypes and social distance (Ando et al., 2011; Della Libera 2023). Seemingly, giving respondents first-hand experiences of acute symptoms of psychosis risks making states of psychosis appear even more strange and disturbing to them.

Another, highly plausible way to increase relatability are contact interventions. Corrigan laid out principles of strategic stigma change through contact [23]: To effectively reduce stigma, contact needs to be targeted to specific situations and audiences, credible in terms of recovery, continuous, and local also in a cultural sense. Seen through the lens of relatability, these principles all seem to increase relatability. Encountering someone who is relatable, because they share biographical, cultural and professional attributes with us, and who have recovered from a seemingly unrelatable state of psychosis, but can explain how they experienced this state themselves, might increase relatability even in such extreme circumstances as acute psychosis [24]. Measuring relatability before and after such interventions could potentially help evaluate the effectiveness of contact and other anti-stigma interventions.

Are there ways to maximise intervention effects on relatability, and, simultaneously on the perception of an us/them continuum? Obviously, we can only hypothesize on that, but would propose three avenues. First, informing about the *overlap* of symptom experiences withcommon, normal experiences might strengthen relatability, and continuum beliefs. Second, stressing the relatable *emotional experience* behind symptoms that are difficult to relate to might show that even strange symptoms are experienced by a person I can relate to. Third, narratives of *recovery* from psychosis, transitioning from a difficult-to-relate to an easy-to-relate state might also increase the notion of a relatable person experiencing states that appear strange and unfamiliar. From the literature on contact interventions [25, 26] we gather that these messages would be most effectively delivered by people with lived experience with a mental illness.

### Can changes in relatability explain the growing gap between attitudes towards depression and schizophrenia?

The rise in biological illness explanations for mental disorders during the 1990s (the “decade of the brain”) has been used as a backdrop to explain why attitudes towards people with schizophrenia have deteriorated [1, 2, 27]. Biological illness explanations have been shown to be associated with greater perceptions of differentness and dangerousness, and in turn, with a greater desire for social distance [28, 29]. Among medical doctors, biological causal explanations decreased, and psychosocial causal explanations increased general empathy toward patients with mental disorders [30], hence a strong focus on neurobiology might as well have decreased relatability among the general public.

A negative effect of biological illness explanations has been shown most consistently for schizophrenia. It does not, however, explain the *improvement* of attitudes towards depression, a disorder that has similarly been increasingly associated with biological illness mechanisms [2]. Arguably, relatability of depression (and probably also other common mental disorders) has increased over the last decades, not the least by messages that normalize the experience of depression, and emphasize the susceptibility of everyone to it [31]. Newspaper analyses show that depression is much more often portrayed in the media than schizophrenia, and reports are much more balanced, including information on therapy and very little reference to crime – quite opposite to schizophrenia, which is much less frequently the subject of media reporting, and if mentioned, is predominantly contextualized with crime, without mention of therapeutic options [32, 33].

## Strengths and limitations

Due to the random assignment, a causal relationships from vignette type on the one side to relatability, us/them continuum beliefs and social distance on the other side can be reasonably inferred. Associations between relatability and social distance and us/them continuum beliefs and social distance, however, are not protected by experimental manipulation and could be challenged with respect to reverse causation or unmeasured confounding. With respect to reverse causation, we have implicitly presumed that respondents first come to judgment concerning whether the described person is someone they can relate to and then decide whether to exclude them from social interaction (social distance). Reverse causation would indicate that a participant first decides whether to exclude a person from interaction and then after making such a judgment decides whether they are relatable or that their situation falls on an us/them continuum. We think this latter scenario is less plausible but also acknowledge that we cannot rule it out. With respect to unmeasured confounding, we would point to the large number of very plausible and impactful confounders we were able to control for (Tables 3 and 4). That and the fact, that associations between relatability and social distance, and us/them continuum beliefs and social distance, are quite large suggests that unmeasured confounders would have to be very large to explain them away. In light of this, the suggestions we made concerning interventions that might directly manipulate relatability might begin to address remaining concerns about a causal relationship between relatability and social distance. Finally, there are other, related constructs that have been evaluated with regard to mental illness stigma and continuum beliefs, namely perceived similarity [34, 35] or likability [36]. Future studies will show how these perceptions add to our understanding of relatability and the difference in reactions to depression and schizophrenia.

*In conclusion*, a large proportion of the difference in reactions to schizophrenia and depression could be traced back to two measures concerned with a perceived gap between the respondents and someone with mental illness, relatability and us/them continuum beliefs. Interventions should be evaluated and tailored with respect to their effect on relatability, and our new measure of relatability could help to evaluate and improve interventions that target the stigma of schizophrenia. Examining relatability and social distance with regard to other mental health conditions like obsessive compulsive disorder or severe substance use disorders will show whether relatability is also relevant for our reactions to other, seemingly incomprehensible mental disorders.

## Electronic Supplementary Material

Below is the link to the electronic supplementary material.

Supplementary Material 1

## Data Availability

The dataset used in this study is available from the first author upon request.
