# Supplementary material for: “Putting yourself in the shoes of others” – Relatability as a novel measure to explain the difference in stigma toward depression and schizophrenia
Source: Soc Psychiatry Psychiatr Epidemiol. 2024 Dec 23;60(8):1883–93. doi: 10.1007/s00127-024-02807-x (PMC12325553; doi:10.1007/s00127-024-02807-x)
Supplement: Supplementary file 1 — Supplementary Material 1 [file 127_2024_2807_MOESM1_ESM.docx]

**“Putting yourself in the shoes of others” – Relatability as a novel measure to explain the difference in stigma toward depression and schizophrenia.**

G. Schomerus ^a^*, J. Kummetat ^a^, M. C. Angermeyer ^b^, B. G. Link ^c^

^a^ Department of Psychiatry and Psychotherapy, University of Leipzig Medical Center, Leipzig, Germany

^b^ Center for Public Mental Health, Gösing am Wagram, Austria

^c^ Department of Sociology, University of California Riverside, Riverside, United States

Online Supplement

**Sampling:**

We conducted an online survey among people living in Germany (18 years and older, n=550), using an established online access panel with quota sampling, with quotas for age, gender, and state proportional to the general population in Germany. After a pretest with 30 respondents, a minimum completion time of 5 minutes was set to ensure the quality of the study. N=965 persons responded to the invite for the survey. N=100 dropped out by not giving informed consent or leaving the survey early, n=136 were excluded by pre-defined quality criteria (failed attention check or below minimum completion time), and n=169 were excluded due to closed regional quotas, i.e. they were from regions where the number of respondents necessary to arrive at a regionally representative sample was already met. This resulted in a final sample of n=550 respondents.

**Instruments: Emotional Reactions, perceived dangerousness, previous contact and general empathy**

We used the nine-item Emotional Reactions towards Mental Illnes (ERMIS) scale (Angermeyer and Matschinger, 2003)to elicit three dimensions of emotional reactions toward the person described in the vignette, asking respondents to give their answers on a five-point scale assessing their agreement or disagreement with the contents of each item. The ERMIS elicits ‘prosocial feelings’ (‘I feel the need to help him/her’, ‘I feel pity’, ‘I feel sympathy for him/her’, alpha 0.70), ‘fear’ (‘I feel uncomfortable’, ‘He/she makes me feel insecure’, ‘He/she scares me’, alpha 0.78) and ‘anger’ (‘I feel annoyed by him/her’, ‘I react angrily’, ‘I am amused by something like that’, alpha 0.82) . Again, we use a mean score from 1-5 for our analyses.

Perceived dangerousness was assessed with a single item, also used in the U.S. General Social Survey (Pescosolido *et al.*, 2019): "In your opinion, how likely is it tha [Lisa] will do something violent towards other people?", answered from 1 ("very likely") to 4 ("very unlikely").

We assessed previous contact with someone with a mental illness with seven items, answered with yes (1) or no (0), which we grouped into an ordinal variable with four levels of contact: 0, No contact. 1, Knows someone; works together or works with ("yes" to either of the following items: “I have seen something about a person with mental illness on TV/cinema/social media”; “I know someone with a mental illness for work/school/leasure time”; “I work with people with mental illness”). 2, Have a friend/family member with mental illness (yes to “I have a friend who has a mental illness”; or “A relative of mine has a mental illness”; or “I live with someone with a mental illness”); and finally 3, self (yes to “I have a mental illness”). We used affirmation of the closest contact to determine the level of contact for each respondent.

We used the German version of the Toronto Empathy Questionnaire (TEQ-D) (Spreng *et al.*, 2009). The measure assesses empathy as one factor with 16 Items, by rating the frequency of habits or manners occurring to the respondent (e.g.: “When someone else is feeling excited, I tend to get excited too.”; “I can tell when others are sad even when they do not say anything.”). Items are scored from 0 “never” to 4 “always” on a five-point Likert-Scale, including seven reverse-coded items (“I am not really interested in how other people feel.“). A sum score is calculated with higher values indicating more empathy. We use the mean score ranging from 0-4.

**Angermeyer MC and Matschinger H**. (2003) The stigma of mental illness: effects of labelling on public attitudes towards people with mental disorder. *ACTA PSYCHIATRICA SCANDINAVICA* **108**: 304-309.

**Pescosolido BA, Manago B and Monahan J**. (2019) Evolving Public Views On The Likelihood Of Violence From People With Mental Illness: Stigma And Its Consequences. *Health Aff (Millwood)* **38**: 1735-1743.

**Spreng RN, McKinnon MC, Mar RA and Levine B**. (2009) The Toronto Empathy Questionnaire: Scale development and initial validation of a factor-analytic solution to multiple empathy measures. *Journal of personality assessment* **91**: 62-71.
